# The Potential of Silver Nanoparticles for Antiviral and Antibacterial Applications: A Mechanism of Action

**DOI:** 10.3390/nano10081566

**Published:** 2020-08-09

**Authors:** Atiqah Salleh, Ruth Naomi, Nike Dewi Utami, Abdul Wahab Mohammad, Ebrahim Mahmoudi, Norlaila Mustafa, Mh Busra Fauzi

**Affiliations:** 1 Centre for Tissue Engineering & Regenerative Medicine, Faculty of Medicine, Universiti Kebangsaan Malaysia, Kuala Lumpur 56000, Malaysia; atqhsalleh@gmail.com (A.S.); ruthmanuel2104@gmail.com (R.N.); nike.dewiutami@gmail.com (N.D.U.); 2 Department of Chemical and Process Engineering, Faculty of Engineering and Built Environment, Universiti Kebangsaan Malaysia, Bangi 43600, Malaysia; awm.ukm@gmail.com (A.W.M.); ebi.dream@gmail.com (E.M.); 3 Department of Internal Medicine, Universiti Kebangsaan Malaysia Medical Centre (UKMMC), Kuala Lumpur 56000, Malaysia; norlaila@ppukm.ukm.edu.my

**Keywords:** silver nanoparticles, antimicrobials, mechanism of action, antiviral, cytotoxicity, nanotechnology

## Abstract

Rapid development of nanotechnology has been in high demand, especially for silver nanoparticles (AgNPs) since they have been proven to be useful in various fields such as medicine, textiles, and household appliances. AgNPs are very important because of their unique physicochemical and antimicrobial properties, with a myriad of activities that are applicable in various fields, including wound care management. This review aimed to elucidate the underlying mechanisms of AgNPs that are responsible for their antiviral properties and their antibacterial activity towards the microorganisms. AgNPs can be synthesized through three different methods—physical, chemical, and biological synthesis—as indicated in this review. The applications and limitations of the AgNPs such as their cytotoxicity towards humans and the environment, will be discussed. Based on the literature search obtained, the properties of AgNPs scrutinizing the antibacterial or antiviral effect shown different interaction towards bacteria which dependent on the synthesis processes followed by the morphological structure of AgNPs.

## 1. Introduction

Silver (Ag) is a chemical element that has provided promising results in various fields such as medicine, electronics, and household applications, e.g., silver sulfadiazine has been used as a standard treatment for burn wounds to prevent the formation of biofilm on the wound area, thus enhancing the wound recovery progress [1]. Silver is a part of transition metals and has been classified as a precious metal due to its decreasing availability. Silver has interesting properties, yet the uses of the materials are limited due to silver instability towards oxygen [2]. Silver metal will oxidize spontaneously when exposed to free oxygen molecules. In these past few years, there has been an unprecedented rise in the application of nanoscience and nanotechnologies which lead to substantial progress in the production of nanomaterials. Thus, it had made possible to produce silver in nanoscale and these emerging nanoparticle products have attracted interest due to their physical, chemical, and biological properties in comparison with their macro-scaled counterparts [3]. These properties are being assessed through various analytical techniques.

Evaluation of silver nanoparticles (AgNPs) involves various types of analytical techniques which includes X-ray diffractometry (XRD) at wavelength (λ) = 1.54056 Å, X-ray photoelectron spectroscopy (XPS) usually between 300–400 eV, Fourier transform infrared spectroscopy (FTIR) in wavelength range of 400–4000 cm^−1^, ultraviolet visible spectroscopy (UV–vis spectroscopy) at the range of 400–500 nm, transmission electron microscopy (TEM), scanning electron microscopy (SEM), dynamic light scattering (DLS) at scattering angle of 173°, and localized surface plasma resonance (LSPR) at wavelength of 300 nm to 1100 nm [4]. These analyses are important to assess the behavior, bio-distribution, and reactivity of these fabricated nanoparticles. Table 1 shows the basic functions of the analytical techniques used for the characterization of AgNPs. However, the result of these analytical techniques depends on the synthesis of AgNPs which also alter the physicochemical properties of the nanoparticles [5]. Recently, experiments with AgNPs have been conducted extensively due to their greater chemical, physical, and biological features compared to their bulk counterparts, such as their size, composition, crystallinity, shape, and structure.

The size, morphology, surface, and particle distribution of nanoparticles have proven to influence the physicochemical properties of AgNPs which can be altered through various synthesis methods, reducing agents, and stabilizers [14]. Size has been the determining factor of biological properties of AgNPs and can be adjusted according to specific application typically in the range from 2 nm until 100 nm e.g., the size of AgNPs for drug delivery application need to be greater than 100 nm to accommodate for the quantity of the drug to be delivered [15]. The toxicity of AgNPs is also dependent on the size of the nanoparticles: the smaller the particles, the higher the toxicity due the higher reactivity and ion release in cells [16]. The shape of an AgNP also influences the toxicity as it can be applied in various types of nanostructures such as nanoplates, nanospheres, nanorods, and flower-like nanoparticles [17]. Moreover, AgNPs are used in antimicrobial applications with proven antimicrobial characteristics of Ag+ ions. These exceptional properties of AgNPs have enabled their use in the fields of nanomedicine, pharmacy, biosensing, and biomedical engineering.

Silver nanoparticles (AgNPs) are already in high demand in various fields which includ health care, medical, and industrial purposes, due to their unique physical and chemical properties. Their unique properties have been influenced by their surface-to-volume ratio which enables modification of their physical, chemical, and biological properties [18]. AgNPs can be synthesized in different types of media—the liquid with different colloidal shapes, and infused into solid materials. The most intriguing properties of AgNPs is their biological activity which influenced by the size distribution, surface chemistry, particle morphology, chemical composition, agglomeration, capping agent, particle response in media, ion release, and the reducing agents used during AgNP synthesis [19]. Understanding the physicochemical properties of AgNPs is crucial as it affects the cellular uptake, infiltration into biological membrane or barriers, the particles distribution, and the therapeutic effects. Therefore, the development of AgNPs that are uniform in morphology and functionality is important for various applications.

Additional insights into the characteristics of AgNPs are discussed in this review. This review presents comprehensive and detailed information on the synthesis of AgNPs by various methods as well as the antibacterial of a AgNP. In addition, the review also focuses on the antiviral effect, application, safety, and limitation of AgNPs.

## 2. Synthesis of AgNPs

**Figure 1:**
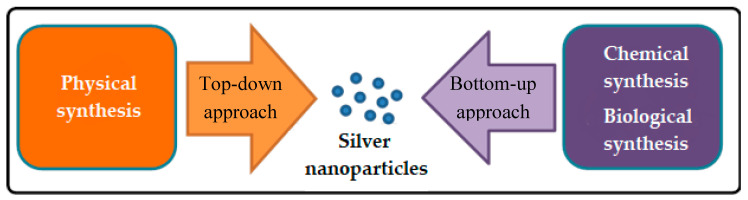
Method of preparing silver nanoparticles.

There are various methods that have been done to synthesize silver nanoparticles (AgNPs) consist of physical, chemical, and biological methods which is also known as green synthesis. Figure 1 shows the simple graphic diagram of the AgNP synthesis overview. Generally, AgNP syntheses are classified as “top-down” and “bottom-up” method. The top-down method is basically the physical method consisting of mechanically grinding the silver bulks while the bottom-up methods consist of chemical reduction, sono-decomposition, and electrochemical methods [20].

### 2.1. Physical Synthesis of AgNPs

Physical synthesis of AgNPs excels in producing a uniform and fair distribution of AgNPs prepared on a thin film due to the absence of solvent contamination. Vapor condensation method and laser ablation are the widely used physical approaches in the synthesis of AgNPs [21]. Vapor condensation method is a two-step method involving the process of evaporation and condensation which carry out in a tube furnace at atmospheric pressure [22]. The range of nanoparticles’ size obtained from this method are in the range of 3 nm to 50 nm. The drawbacks of physical synthesis of AgNPs are it is a time-consuming process and require a high amount of energy which can also cause the environmental temperature to rise. It is also difficult to achieve thermal stability due to high energy requirement e.g., a typical tube furnace requires high power consumption more than several kilowatts and preheating time of several minutes in order to reach a stable operating temperature [23]. Therefore, there have been numerous alternatives to physical methods instead of implementing the evaporation and condensation method.

Another example of the physical synthesis of AgNPs is done by the laser ablation method that results in the average size of AgNPs between 10 nm to 30 nm. In brief, the process includes laser ablation of bulk metallic silver salts or materials in solution phase [24]. Pure colloidal AgNPs can be produced due to the absence of chemical reagents in the solvents. However, this method is dependent on the laser wavelength, fluency, ablation duration, liquid medium (with or without surfactants), and the duration of laser pulses in femto-, pico-, and nano-second regime). Zhang and his fellow researcher have stated that there is more frequent usage of Femtosecond Laser Ablation in Liquids (fs LAL) due to its efficiency in controlling the particle size and material ablation [25]. Silver nanoparticles that have 13 to 18 nm particle size were prepared through fs LAL at 400 nm.

### 2.2. Chemical Synthesis of AgNPs

Chemical synthesis has been widely used as an alternative method in preparing silver nanoparticles in colloidal dispersion. The chemical method of preparing AgNPs involves water or organic solvents in order to reduce the silver metal salts. This process involves three components which are reducing agents, metal precursors, and stabilizing or capping agents. Reducing and capping agents is an important step in producing AgNPs because it can alter the characteristics of AgNPs according to its applications and purposes e.g., AgNPs in dental applications required particles size of 15–20 nm. The selection of reducing agents is a rate-limiting process used to achieve the specific characteristics of AgNPs in terms of size, physicochemical properties as well as prevent the coagulation of AgNPs [26]. The disadvantage of preparing AgNPs using chemical synthesis is that the materials used are toxic and hazardous [27]. The nanoparticles also exposed to surface contamination due to chemical sedimentation and the uses of chemical reducing agents are harmful. However, chemical synthesis is able to provide higher yields, easy to reproduce AgNPs, and it has lower cost in comparison with the physical methods.

The productions of silver nanoparticles with different morphologies and sizes have already been reported in various articles. Table 2 shows the simplified version of the chemical synthesis of AgNP. Wang et al. 2004 have conducted an experiment to reduce silver nitrate into nanoparticles in the presence of polyvinyl pyrrolidone (PVP) which result in the formation of AgNPs with a size range of 20 to 70 nm [28]. The experiment uses glucose as reducer and sodium hydroxide as an accelerator of the chemical reaction. There are two possible ways of silver reduction in an aqueous PVP solution. The first equation involves the reaction of the silver ion with PVP generating complex ion (Equation (1)). Then, the hydroxyl ion undergoes nucleophilic addition reaction with glucose then reduces silver ion (Equation (2)). Meanwhile, the third equation involves the reaction of silver ions with hydroxyl ion producing Ag_2_O (Equation (3)) which then reduced by glucose causing generation of silver particles (Equation (4)). The reaction between glucose and silver ion in PVP solution can be written as follows:

Ag^+^ + PVP → Ag(PVP)^+^ (1)

CH_2_OH(CHOH)_4_CHO + 2[Ag(PVP)]^+^ + 2OH^−^ → CH_2_OH(CHOH)_4_COOH + 2Ag(PVP) + H_2_O (2)

2Ag^+^ + 2OH^−^ → Ag_2_O + H_2_O (3)

Ag_2_O + CH_2_OH(CHOH)_4_CHO + 2PVP → CH_2_OH(CHOH)_4_COOH + 2Ag(PVP) (4)

### 2.3. Biological Synthesis of AgNPs

To overcome the disadvantages of the chemical synthesis of AgNPs, biological methods have been preferred and are thus well studied as an alternative method in preparing silver nanoparticles. The application of biologically-mediated synthesis is proven to be cost-effective, simple, and environmentally friendly as it does not use hazardous or toxic chemicals [37]. This approach has been given much attention as it produces the high yield of AgNPs and it replaces chemicals with bacteria, plant extracts, and small biomolecules such as amino acids. The green synthesis involves three important factors: (1) solvent, (2) reducing agents, and (3) non-toxic material [38]. The advantages of this method are the availability of biological resources used as reducing agents, the high stability in a short production time and the readily soluble nanoparticles.

A previous study had extensively discussed the differences between chemical and biological methods of producing AgNPs [39]. The chemical synthesis was performed by the following method suggested by Fang, Zhang, and Mu using sodium citrate as the reducing agent while the biological synthesis was done by using white-rot fungi *Pycnoporus* sp. [40]. The study concluded that the synthesis of AgNPs by chemical reduction is dependent on the reducing agents and stabilizer which controls the particles aggregation and other parameters while green synthesis dependent on the enzymes secreted by the fungi. The production of AgNPs by fungi occurs in these two steps: (1) entrapment of the silver ions at the surface of the fungi and (2) the reduction of AgNPs by the enzyme secreted from fungal system [41]. Table 3 shows the simplified information on the biological synthesis of AgNPs. These synthesized nanoparticles also show positive antibacterial properties where its mechanism of action will be explained in this review.

## 3. Mechanism of Action

Silver has become one of the most widely studied oligodynamic materials due to its range of bactericidal activities, effectiveness, low toxicity, and various application as disinfectant. The oligodynamic effect is the biocidal effects of metals, especially heavy metals, which could occur in low concentrations of heavy metals. Silver nanoparticles (AgNPs) have proven to have oligodynamic effect due to their large surface areas with its ability to bind with bacterial biomolecules, the ability to penetrate the cells, generation of reactive oxygen species (ROS) and free radicals, and act as modulators in signal transduction pathways of microorganisms [54]. The broad-spectrum of oligodynamic materials, in particularly AgNPs, could enhanced the development of nanotherapeutics such as antibiotics have received a lot of attention.

The surface modification of AgNPs can also affect the oligodynamics effect of nanoparticles as surface modification can stabilize these nanoparticles e.g., modification of AgNPs with polyimide. There is a study that has proven half encapsulation of AgNPs with loosely polyimide can increase the antibacterial activity of these nanoparticles as well as improve the silver stability [55]. Coating with polydopamine and hydrofluoric acid etched glass spheres (PDA-HF/GSs) have shown strong adhesion towards *E. coli* and *Bacillus* [56]. Thus, preventing the formation of biofilm. Modification of AgNPs by incorporating metal oxide through a solution casting method exhibited a uniform dispersion of nanoparticles. These modified nanoparticles also inhibit the growth of *Escherichia coli* and *Staphylococcus aureus* as well as prevent formation of film [57].

The antibacterial effects of AgNPs are more complex compare with other metallic nanoparticles and shows lower toxicity toward cells. Some metallic nanoparticles show higher antibacterial activity than AgNPs but are toxic towards cell, e.g., copper nanoparticles (CuNPs). CuNPs show greater ability to inhibit bacterial growth than AgNPs as their mechanism of action involve rupture of bacterial membrane and electron transfer by photocatalytic process [58]. Even though CuNPs have higher antimicrobial activity, they are more instable and have higher possibility to convert into CuONPs through oxidation process. Meanwhile, gold nanoparticles (AuNPs) have lower ability to inhibit bacterial growth in comparison with AgNPs as they do not exhibit apparent intrinsic antibacterial activity. Their mechanism of action only involve adhesion on the bacterial surface through electrostatic forces [59]. This mechanism of action is dependent on the size of nanoparticles, the smaller the nanoparticles, the smaller minimum inhibition concentration (MIC) [60]. On the other hand, antiviral properties of other metallic nanoparticles work the same mechanism as AgNPs. The antiviral activity of metallic nanoparticles consists of competitive binding with cell receptor and rupture of viral envelope [61]. Therefore, the antibacterial and antiviral properties of AgNPs are further discussed below.

### 3.1. Antibacterial Properties of AgNPs

**Figure 2:**
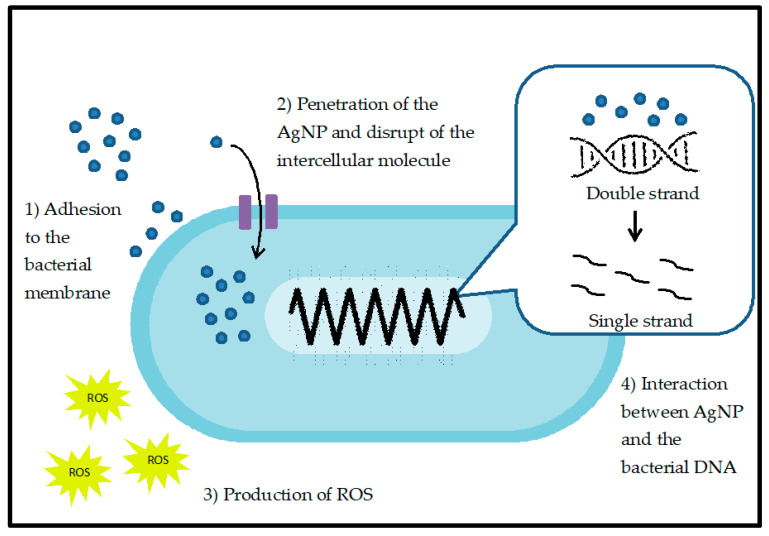
Antibacterial activities of silver nanoparticles.

Silver nanoparticles (AgNPs) have caught the attention of researchers due to its broad spectrum of antifungal and antibacterial properties. New developments of antibacterial products or agents are in dire need due to resistance of microbial towards antibiotics through mutation. Moreover, the low reactivity of AgNPs compared to the silver ions made them suitable for clinical and therapeutic application [62]. There are four known antimicrobial actions of AgNPs: (1) adhesion towards the surface membrane of microbial. (2) Penetration of AgNPs into the cells and cause disruption of biomolecules and intracellular damage, (3) induce cellular toxicity by generating ROS which trigger the oxidative stress of cell, and (4) disrupt the signal transduction pathways of the cells [63]. Figure 2 shows the overall mechanism of action in bacteria.

When the microbes are exposed to AgNPs, the nanoparticles tend to stick or adhere to the cell wall or membrane of microbes due to the electrostatic attraction between the positive charge of silver ions which is generated from oxidation of AgNPs and the negatively charged cell membrane of microorganisms [64]. AgNPs also have strong affinity towards the sulfur-containing proteins in the microbial cell wall. The attachment of AgNPs towards the membrane of microbes causes irreversible morphological changes in the structure of cell membrane [65]. This can also cause a loss in the integrity of lipid bilayer and the permeability of the cell membrane. Alteration in cell structure can cause increased permeability of the cell membrane which in turn affects the cell ability to regularly regulate activity. For instance, the release of the silver ions by the nanoparticles will alter transport and releases of potassium ions (K+), thus affecting the transport activity of cells. Increase in cell membrane permeability may also cause loss or leakage of cellular contents such as cytoplasm, proteins, ions, and cellular energy reservoir, ATP, which can induce the ghost cell effect of the microorganisms. The ghost cell effect in bacteria occurs when the expulsion of the cell or microbial contents leaving a hollow envelope of the microorganisms [66]. Transmission electron microscopic (TEM) images have illustrated that AgNPs affect the integrity of Gram-negative bacteria (*E. coli* and *S. typhimurium*) membrane by depolarization and destabilization of membrane [67].

As the AgNPs bind to the surface of the microbial membrane, they can also penetrate the cells and affect important biomolecules and cellular activity. AgNPs can enter the bacterial cells through a water-filled channel called porins in the outer membrane of Gram-negative bacteria such as *E. coli*. After penetration of AgNPs into the cells, these nanoparticles will start to bind with cellular structures and biomolecules such as proteins, lipids, and DNA, thus damaging the internal structure of the bacteria. The silver ions that have been released into the environment will bind to negatively charge protein which altering the protein structurally and eventually result in deactivation the proteins. The result from Li et al. (2010) proved that the AgNP inhibits the respiratory chain dehydrogenase by conversion of various enzymes such as glycerol-3-phosphate dehydrogenase into dihydroxyacetone in *S. aureus* (Gram-positive bacteria), and thus interfere with normal growth and metabolism of the bacteria cells [68]. Additionally, AgNPs can interact with bacterial DNA causing denaturation of DNA and interrupt the cell growth of the microbes [69]. AgNPs can decrease the stability of DNA structure by electrostatic repulsion due to the DNA and AgNPs having the same polar charge [70]. Sadoon et al. (2020) have shown that silver ions can interact with DNA thus causing hybridization of doubles-stranded DNA which involves dissociation of double strands into single strands by disrupting the H-bonds of DNA strands [71].

Another mechanism of action in AgNPs is the production of reactive oxygen species (ROS) which normally causes cellular oxidative stress in microbes. ROS is a general term for oxygenated compounds that involved in various cellular biological events such as superoxide, hydrogen peroxide and hydroxyl radicals. The antibacterial potential of AgNPs is usually related to the ability of nanoparticles to produce ROS and free radicals and eventually increase the oxidative stress in cells. Productions of intercellular ROS have become the most important indicator towards toxicity related to nanoparticles as they may induce lipid damage, leakage of cellular biomolecules and eventually lead to cell apoptosis [72]. Song et al. (2018) have found out that treatment with curcumin and AgNPs may enhance the antibacterial properties towards *B. sublitis* and *E. coli* by increasing the formation of ROS which leads to membrane damage and is followed by cell death [73]. The TEM results of the study described that the rupture of the cell membrane in the treated group with curcumin and AgNPs. Zhang et al. (2019) suggested that the production of ROS is primarily dependent on the concentration or dose of AgNPs [74]. The study has shown that the production of ROS was dependent on the size of silver nanoparticles where AgNPs with a concentration of 10 mg/L cause the highest level of ROS generation in *A. vinelandii* and *N. europaea* which were treated with 10 mg/L and 50 mg/L of AgNP.

The mechanism of signaling pathway depends on the phosphorylation and dephosphorylation cascade of protein or enzymes which are essential for cellular activity and bacterial growth. Due to unique physicochemical properties of AgNPs, there is a possibility for these nanoparticles to act as modulators of signal transduction in microbial cells [75]. Jena et al. (2020) had demonstrated that the gold-silver nanoparticles able to mediate apoptosis of bacteria cell by disrupting the bacterial actin cytoskeletal network [76]. The result shows that the nanoparticles affect the actin cytoskeleton MreB causing morphological changes in the bacterial shape thus increase the fluidity in the membrane which follow by rupture of cells. MreB, an actin homologue, plays an important role in regulating the localization of cell shapes and cell survival. Table 4 shows the antibacterial studies of AgNPs and their mechanism of action. As these nanoparticles show effective oligodynamic effects on bacteria, there also some studies that shown their effects towards viruses.

### 3.2. Antiviral Properties of AgNPs

The recent outbreaks of an infectious disease caused by a newly evolving pathogenic virus, which has developed resistance towards available antiviral drugs, has encouraged numerous researchers in the search for new antiviral agents. The viral disease depends on the entry and attachment of the virus onto the host cells by binding of viral surface components with ligands and proteins on the cell membrane. The best strategy in developing new antiviral agents is to interfere with the interactions of virus ligand and cell membrane, thereby blocking the attachment and entry of the virus into the cells. By considering the metal nanoparticles mechanism of action in microbes, silver nanoparticles (AgNPs) have become one of the strongest candidates as antiviral agents. The broad attack ranges of AgNPs towards its target have made resistance of microbes towards these nanoparticles become futile. 

**Figure 3:**
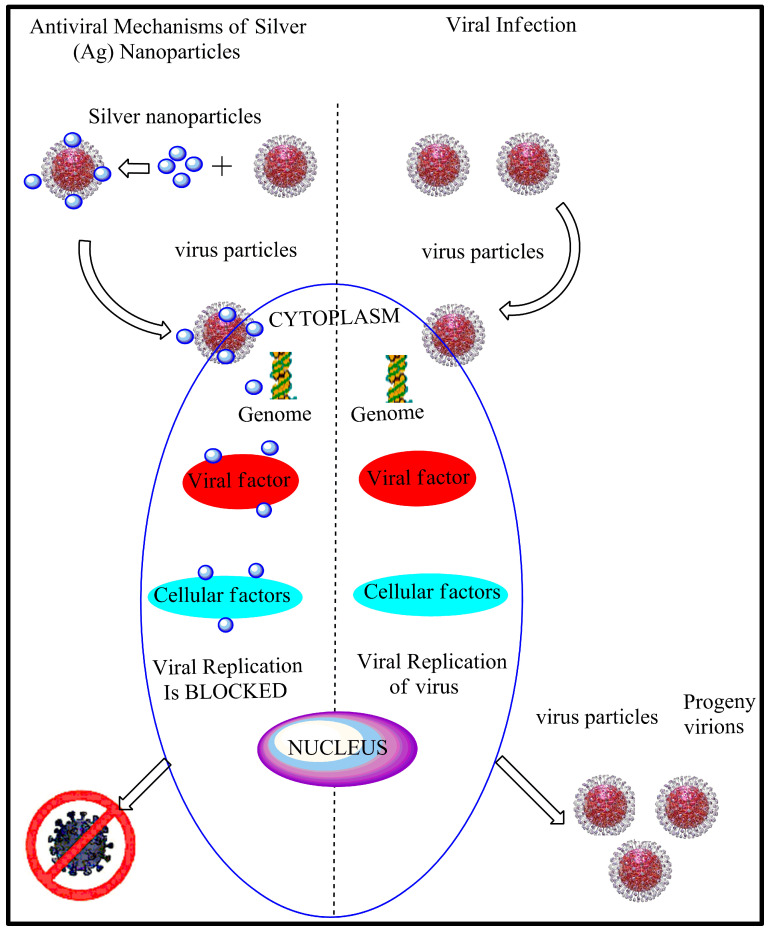
The antiviral activities of silver nanoparticles.

Figure 3 shows a diagram that generally explains the AgNP mechanism of action towards a virus.

There are a few types of research on the effects of AgNPs towards viruses. However, the details of the interaction are limited. The complexity of virus structures can contribute to the limited knowledge of nanoparticles mechanism towards the viruses. There are two ways the AgNP interacts with the pathogenic virus: (1) the AgNP will bind to the outer coat of the virus thus inhibit the attachment of virus towards cell receptors and (2) the AgNP will bind to the DNA or the RNA of the virus thus inhibiting the replication or propagation of the virus inside the host cells. Table 5 show the antiviral properties of AgNPs towards different types of pathogenic virus. Understanding these nanoparticle’s mechanism of action towards different types of viruses could develop new viral therapy by using nanotechnologies. 

A virus often triggers a cell’s apoptosis when it undergoes the viral replication process. The apoptosis of cells causes the host to have severe symptoms and could cause fatality if not treated. Mitochondrial-mediated apoptosis pathway is an example of signaling pathway that could induce cell apoptosis involving depolarization of mitochondrial membrane potential (MMP). This pathway involves 2 different protein which are Bax and Bcl-2. Bax is the pro-apoptotic protein causing permeabilization of mitochondrial membrane which allow proteins in mitochondrial intermembrane space to escape to the cytosol to induce cell apoptosis while Bcl-2 is the anti-apoptotic protein which inhibit Bax mechanism [93]. Lv et al. 2014 have tested different silver nanomaterials consisting of AgNPs, silver colloids, and 2 different kinds of silver nanowires against transmissible gastroenteritis virus (TGEV) [94]. The article had done pre-clinical tests by infecting TGEV to swine testicle (ST) cells and the results shows that TGEV is able to induce mitochondrial-mediated apoptosis pathway by increasing the Bax levels in the cells. The silver nanomaterials used in this article have shown to inhibit the initiation of TGEV infection by binding to the surface protein, S glycoprotein. The article also suggested that the silver nanomaterials able to alter the structure of surface proteins thus inhibit their recognition and adhesion towards host receptor. The antiviral activities of these nanomaterials could lower the risk of infection or might as well prevent an epidemic viral disease such as COVID-19.

**Figure 4:**
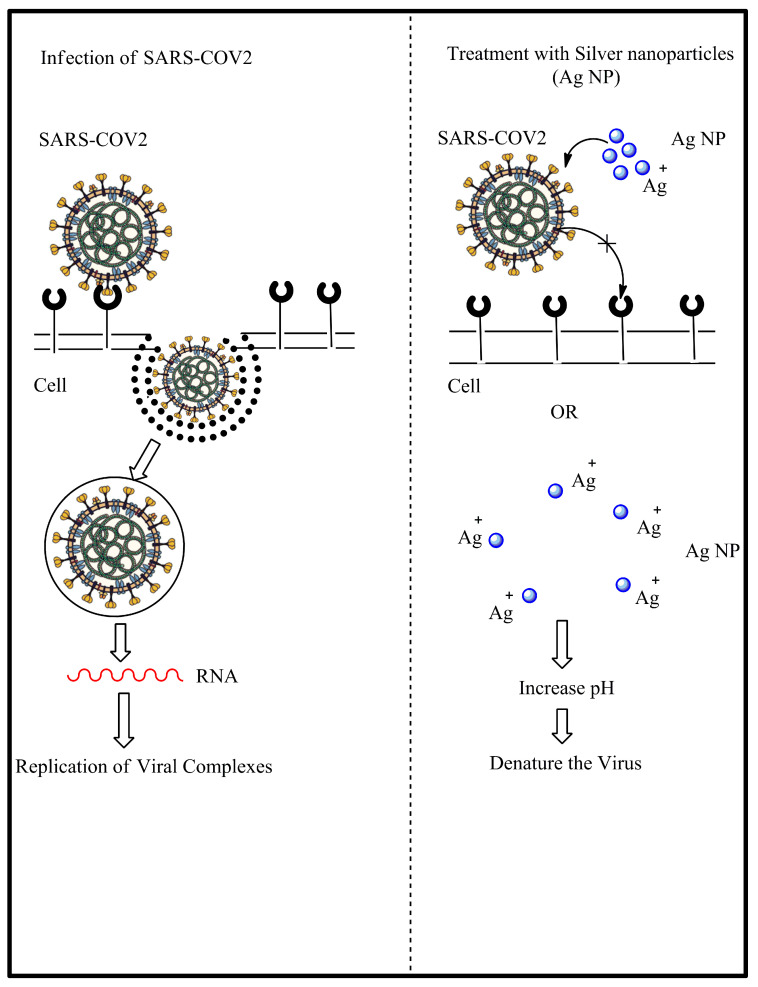
The proposed possible mechanism of action towards severe acute respiratory syndrome coronavirus 2 (SARS-CoV 2).

The Coronavirus disease 19 pandemic, commonly known as COVID-19 is caused by severe acute respiratory syndrome coronavirus 2 (SARS-CoV-2). The coronaviruses belong to family *Coronaviridae* in *Nidovirales* order. *Coronaviridae* often associate with infection in the upper part of respiratory system and the gastrointestinal tract of host. The term “corona” represents morphological structure of the protein spikes on the outer surface of the virus which resemble a crown. The size of coronaviruses are in the range of 65 to 125 nm in diameter which only contain a single-stranded RNA (size range from 26 to 32 kbs in length) as nucleic material [95]. With the recent outbreak of new viral diseases such as COVID-19 that has reached over 8 million cases worldwide, the application of AgNPs could be implemented as treatment. There already is an opinion letter on using silver nanoparticles as the antiviral therapy to treat COVID-19 patients with minimum side effects [96]. The hypothesis was the AgNPs will bind to the spike glycoprotein of the virus thus inhibiting the binding of the virus towards the cells and the release of silver ions can decrease the environmental pH of respiratory epithelium (where the COVID-19 virus usually reside) to become more acidic which is hostile towards the virus. Figure 4 shows the overall hypothesis of AgNPs antiviral activities against SARS-CoV 2. Both antiviral and antibacterial action of AgNP can be used to contribute to various industries, especially health care, where they can be used to combat infection problems worldwide.

## 4. Application of AgNPs

Silver nanoparticles (AgNPs) have been useful in different industrials, e.g., textiles, biomedicines, foods, and electronic applications, due to their unique physicochemical and biological properties. AgNPs are applied as antibacterial agents in disinfectants to water treatment. Moreover, the uses of AgNPs in the textile industry have gained quite a lot of attention due to the greater ion release and increasing catalytic activity as a result of the large surface area per mass of AgNP. The AgNPs are embedded into the fabric by two primary methods: (1) inserting the substrates with pre-formed nanoparticles and (2) inserting the substrates into a solution with silver salts and treating with reducing agents in order to convert silver salts into AgNPs [97]. A silver nanocoated fabric shows a homogeneous distribution of AgNPs and excellent antibacterial activity [98]. Next, the applications of silver in medicine have already been applied for a few decades. AgNPs have been applied as wound dressing products as it has been proven to significantly reduce the wound healing time while simultaneously prevent infection in the wound site [99]. Silver nanoparticles embedded in bacterial cellulose are able to perform the bactericidal activity on a Gram-negative bacterium (*E. coli*) and non-toxic to epidermal cells [100]. Other than nanoparticles on their own, functionalized AgNPs also have been deemed useful in various industries.

Conjugations of AgNPs with other types of polymers have shown beneficial results especially stabilizing the nanoparticles as well as providing a chemically stable system [101]. Functionalized AgNPs have shown to be useful in the medical industry as it helps in drug delivery or enhancing medical devices. AgNPs that have been functionalized through the process of PEGylation are some of the most commonly used functionalized AgNPs. PEGylation is amalgamation of both covalent and non-covalent of polyethylene glycol (PEG) polymer chain with the nanoparticles, in this case, AgNPs are coated with polyethene glycol (PEG) to improve the drug delivery to targeted cells. PEG coats able to prevent aggregation of nanoparticles as well as increases the circulation time for drug delivery. Hajtuch and fellow researchers have conducted an experiment on functionalized AgNPs with reduced glutathione (GSH), PEG, and lipoic acid (LA) to investigate the effects of AgNPs on human blood platelets [102]. The study has proven that these functionalized AgNPs are able to reduce platelet aggregation which most likely is caused by protein interaction of platelet surface and AgNP.

Functionalization can also increase the antimicrobial activity of these nanoparticles. Addition of platinum can enhance the formation of silver ions through galvanic action thus enhance the efficiency of anti-bacterial activity without affecting cytoxicity [103]. Galvanic action is an electrochemical process where two different metals creating an electron conductive path in the presence of electrolyte, in this case, the electron flow to the AgNP cause increased formation of silver ions. Compounds such as acetic acid can also increase the antimicrobial activity of the nanoparticles [104]. In wound healing application, it is important to control the release of silver ions because excessive formation of silver ions can cause oxidative stress in the cells as the production of reactive oxygen species increases. There are various studies indicating that immobilization of AgNPs has more advantages in comparison with unbounded nanoparticles. Wu and co-workers have used bacterial cellulose to immobilize AgNPs causing even distribution across the cellulose, stabilization, and minimizing the toxicity of the nanoparticles [100]. While, Balamurugan and co-workers have used a bioglass system incorporated with silver through a process called sol-gel in order to minimize release of silver ions [105]. However, it is beneficial to learn the hazards of AgNP to prevent problems from occurring during application of AgNPs.

## 5. Safety of AgNPs

Silver nanoparticles (AgNPs) have been very useful towards many fields especially the medical field due to their wide range of applications in disinfection. However, there have been a couple of issues regarding AgNPs that are still highly debatable between researchers involving the toxicity and the environmental impact of AgNPs. These nanoparticles are viewed as potential hazards due to its ability to induce cytotoxic mechanisms such as production of reactive oxygen species (ROS), DNA damage, and heighten pro-inflammation progress in cells. Due to the unique physicochemical properties of AgNPs, especially production of reactive oxygen (ROS), these nanoparticles can cause sublethal adverse effects when the concentration or production of ROS is not controlled properly [106]. There a study has shown the toxicity of AgNPs by using in vitro model. Mao and co-workers had proven that AgNPs induced high production of ROS causing a defect in formation of larvae which was activated through oxidative stress in *Drosophila melanogaster* [107]. There was also the case where a patient was diagnosed with argyria due to over-consumption of colloidal silver [108]. Argyria is a rare skin condition which causes cutaneous discoloration due to intake or exposure to silver. Another issue regarding nanoparticles is the effect of AgNPs on the environment. The impacts of AgNPs towards the environment remain unclear due to limitation in current available technologies. However, Xu and fellow researchers stated that AgNPs have a negative impact on the soil microbiome in the long run [109]. This shows that AgNPs also have limitations.

## 6. Limitations of AgNPs

Silver nanoparticles (AgNPs) are known for their advantages in physicochemical properties in comparison with their bulk structure. However, these nanoparticles have their own limitations whereas one of the limitations is fast reaction with oxygen called oxidation. AgNPs are very sensitive with oxygen as the silver ion will bind to oxygen and form a strong bond called an ionic bond. The formation of this ionic bond will alter the structure of nanoparticles, thus changing the physicochemical properties of nanoparticles. The oxidized AgNPs also reduce the antibacterial properties of AgNPs due to its dependence towards silver ions [110]. Another limitation in AgNPs is the tendency of these particles to be aggregated. There also a study performed by Menazea (2020) that have tested AgNPs in different medium and the result shows that these nanoparticles tend to aggregate in dimethylformamide and tetrahydrofurane solutions which are an organic medium [111].

## 7. Conclusions

The occurrence of nanoparticles, particularly metallic nanoparticles, have been deemed very useful in numerous fields and have been acknowledged worldwide. This inclusive research on silver nanoparticles has been discussed in this review to have a better understanding on the characterization of physicochemical properties, synthesis, mechanisms of action, application, safety, and limitation of AgNPs. The unique physicochemical properties of the AgNP are dependent on various parameters of AgNPs such as size, surfactant, and structure morphology of these nanoparticles. Even though there are various methods in producing AgNPs, biological method or green synthesis have high yield and biocompatibility as it uses natural agents and nontoxic chemicals. In this review, we provide an inclusive discussion on the mode of action of AgNPs that have drawn attention as antimicrobial agents covering the antibacterial and antiviral properties of AgNPs towards various types of pathogenic viruses. Topics on the safety and limitation of AgNPs were also highlighted in this review where the aggregation and cytotoxicity of these nanoparticles have becoming the main concerns of AgNP applications. Lastly, research on a better understanding in cytotoxic mechanisms of AgNPs towards the environment by breaking the technologies limit could benefit the future prospects of AgNPs.

## Figures and Tables

**Table 1:** Analytical techniques and their basic functions in the characterization of silver nanoparticles.

Analytical Technique	Functions	References
X-ray diffraction	Measure the degree of crystallinity at the atomic scale. Used to analyze the structure of nanoparticles, particle sizes, for compounds identification, and to determine structure imperfections in the structures. The analysis depends on the formation of diffraction patterns	[6]
X-ray photoelectron spectroscopy	Determine the electronic states by atoms which include the oxidation state, and electron transfer in the nanoparticles. Estimate the empirical formulae by surface chemical analysis. Characterize the nanoparticles’ surface in the liquid forms.	[7]
Fourier transform infrared spectroscopy	Characterize various chemical bonding in nanomaterials.	[8]
UV–vis spectroscopy	Evaluate the stability and characteristics of AgNPs. Absorption of AgNPs depends on the dielectric medium, particle size, and the chemical environment. Size depends on surface plasmon for metal nanoparticles ranging from 2 to 100 nm.	[9]
Transmission electron microscopy	Measure of particle size, morphology, and size distribution. Provide better spatial resolution compared to SEM.	[10]
Scanning electron microscopy	Evaluate the morphology of AgNPs. Histogram obtains from images. Manually measure and count the particles or using specific software.	[11]
Dynamic light scattering	Measure nanoparticles size. Evaluate their stability over time in suspension at different pH and temperature conditions.	[12]
Localized surface plasmon resonance	Determine spatial oscillation of non-excited or excited (near-visible light) electron. Evaluate the molecular interaction on the surface of a nanoparticle. Depends on several factors: particle’s size and shape, electronic properties, dielectric media, and temperature	[13]

**Table 2:** The chemical synthesis of AgNP.

Reaction	Results	References
Turkevich method: Reduction of silver nitrate with sodium citrate	The particles size of 14 nm with a mean diameter of 10 nm	[29]
Reduction of silver nitrate with a mixture of hydrazine hydrate and sodium citrate as reductants; sodium dodecyl sulfate as a stabilizer	Colloidal solution with the particles size range of 8 to 50 nm with a mean diameter of 24 nm	[30]
Reduction of silver nitrate with a mixture of two different reducing agents which are tannic acid and sodium nitrate	Combination of reducing agents able to produce monodisperse spherical silver nanoparticles in 5 to 140 nm	[31]
Reduction of silver nitrate by sodium borohydride in presences of sodium dodecyl sulfate as a stabilizer Ag^+^ + BH_4_^−^ + 3H_2_O → Ag^o^ + B(OH)_3_ + 3.5 H_2_	Formation of colloidal silver nanoparticles with particles diameter in a range from 30 to 40 nm	[32]
Reduction of silver nitrate with dextrose as reducing agent in presence of Na^+^-carrying poly[γ-glutamic acid] (PGA) 2Ag^+^ + 2OH^−^ → Ag_2_O + H_2_O Ag_2_O + 4NH_3_ + H_2_O → 2[Ag(NH_3_)_2_]^+^ + 2OH	Formation of silver nanoparticles with an average size of 37.3 ± 5.5 nm for 0.5 wt% PGA-AgNP and 17.3 ± 3.4 nm for 2 wt% of PGA-AgNP	[33]
Reduction of silver nitrate with aniline in the presence of cetyltrimethylammonium bromide (CTAB) Ph-NH_2_ + Ag^+^ → Ph-NH_2_-Ag^+^ Ph-NH_2_-Ag^+^ → Anilino radical + Ag^o^ + H_2_O	Formation of spherical nanoparticles in size range from 10 to 30 nm and wide size distribution	[34]
Reduction of silver nitrate with trisodium citrate 4Ag^+^ + C_6_H_5_O_7_Na_3_ + 2H_2_O → 4Ag^o^ + C_6_H_5_O_7_H_3_ + 3Na^+^ + H^+^ + O_2_↑	Formation of silver nanoparticle with particle size range from 5 to 100 nm	[35]
Reduction of silver nitrate with two different reducing agents which are ethylene glycol (EG) and glucose in the presence of poly[N-vinylpyrolidone] (PVP) as a stabilizer 2AgNO_3_ + R-CHO + 2 NaOH → 2Ag + R-COOH + 2NaNO_3_ + H_2_O	Spherical silver nanoparticles with particle size range from 10 to 250 nm	[36]

**Table 3:** The green synthesis of silver nanoparticles (AgNPs).

Substances	Results	Reference
(a) Plants		
*Artemisia nilagirica* extract	The size diameters of the nanoparticles are in the range of 70 to 90 nm and the size distribution in the range of 2 to 4 keV. There was no impurity found.	[42]
Leaves extract of *Catharanthus roseus*. Linn. G. Donn	The size of nanoparticles is 27 ± 2 nm with zeta-potential of −63.1 mV which indicate good dispersity and stability.	[43]
*Boerhaavia diffusa* plant extract	The average particles size is 25 nm with cubic morphology of silver nanoparticles.	[44]
Ethanolic extract of *Terminalia fagifolia* Mart.	Formation of spherical or polygonal silver nanoparticles with a size range of 66 to 81 nm with high polydispersity.	[45]
*Rosemary* leaf aqueous extract	The silver nanoparticles are in a spherical shape with a diameter size of 14 nm with high purity.	[46]
*Butea monosperma* (BM) leaf extract	Formation of triangular and spherical nanoparticles with a size range of 20 to 80 nm.	[15]
Curcumin:hydroxypropyl-β-cyclodextrin (CUR:HPβCD)	Formation of spherical silver nanoparticles with an average size of 42.71 ± 17.97 nm and homogeneous dispersion of nanoparticles.	[47]
(b) Fungus		
Biosorption by *Aspergillus flavus*	Production of monodisperse silver nanoparticles with an average particle size of 8.92 ± 1.61 nm and size distribution about 1000 nanoparticles.	[48]
*Cladosporium cladosporioides*	Formation of high-density silver nanoparticles with average size of 24 nm with uniform dispersion.	[49]
*Guignardia* sp.	The size of silver nanoparticles in the range of 5 nm and 20 nm with fairly monodisperse nature.	[50]
(c) Bacteria		
*Bacillus* sp.	Development of silver nanoparticles with a size range of 5 to 15 nm observed in the periplasmic space of bacterial cells.	[51]
*Bacillus subtilis*	The average size of silver nanoparticles produces is 6.1 ± 1.6 nm.	[52]
*Escherichia coli*	The process yielded an average size particle of 50 nm with a uniform distribution at 50 nm.	[53]

**Table 4:** The antibacterial properties of AgNPs.

Bacteria	Type of Bacteria	Silver Nanoparticles Size	Mechanism of Action	References
*Pseudomonas aeruginosa*	Gram-negative	Average particles size of 45 nm	Interaction with ROS and attachment of AgNPs at microbial cell wall	[77]
*Escherichia coli* AB1157	Gram-negative	Average mean diameter 8.3 ± 1.9 nm	Damage the cellular DNA by influencing the base excision repair system	[78]
*Staphylococcus aureus* ATCC25923	Gram-positive	Average size of 3.91 nm, 2.29 nm, and 1.59 nm	Destruction of microbial cell membrane and rise of ROS concentration	[79]
*Escherichia coli* ATCC25922	Gram-negative			
*Escherichia coli* DH5α	Gram-negative	Average size of 30 nm	Accumulation of AgNPs in the cell wall and cell membrane of bacterial cell	[80]
*Bacillus* *Calmette-Guérin*	Acid-fast Gram-positive			
Multidrug resistant *Escherichia coli* (MC-2)	Gram-negative	Average size of 18 ± 3 nm	Disruption of cell membrane through formation of ROS	[81]
Multidrug resistant *Staphylococcus aureus* (MMC-20)	Gram-positive			
*Proteus* sp.	Gram-negative	Average size of 38 nm	Cell wall ruptured and inhibit DNA replication thus inhibit the bacterial growth	[82]
*Klebsiella* sp.	Gram-negative			
*Staphylococcus aureus*	Gram-positive	Size of nanoparticles should be lower than 100 nm. The articles do not mention the size of AgNPs	Oxidative stress which cause alteration in kynurenine protein. Activation of kynurenine pathways thus inhibit the bacterial growth	[83]
*Escherichia coli*	Gram-negative			
*Pseudomonas aeruginosa*	Gram-negative			
*Bacillus subtilis*	Gram-positive			
*Klebsiella pneumoniae*	Gram-negative			

**Table 5:** The antiviral properties of AgNPs.

Virus	Family	Silver Nanoparticles Composition	Mechanism of Action	References
Herpes simplex virus type 2 (HSV-2)	*Herpesviridae*	Tannic acid-modified silver nanoparticles (13 nm)	Interact with viral glycoproteins thus interfere with cell attachment	[84]
Bacteriophage MS2	*Leviviridae*	Magnetic hybrid colloid silver nanoparticles (15 nm)	Damage proteins of the viral coat	[85]
Murine novovirus	*Caliciviridae*			
Herpes simplex virus type 1 and type 2 (HSV-1 & HSV-2)	*Herpesviridae*	Mycosynthsized silver nanoparticles (4–31 nm)	Block interaction of virus and cells	[86]
Human parainfluenza virus type 3 (hPIV3)	*Paramyxoviridae*			
Human immunodeficiency virus (HIV)	*Retroviridae*	PVP-coated silver nanoparticles (30–50 nm)	Inhibit the interaction between gp120 and cell membrane receptors	[87]
H1N1 influenza A	*Orthomyxoviridae*	Chitosan-coated silver nanoparticles (3.5, 6.5, and 12.9 nm)	Inhibit the viral contact with host cells and interaction of silver nanoparticles with viral glycoproteins	[88]
Poliovirus		Pure silver nanoparticles (7.1 nm)	Bind with the viral particles thus prevent binding with host receptor and inhibition of viral proteins	[89]
Respiratory syncytial virus (RSV)	*Paramyxoviridae*	PVP-coated silver nanoparticles (10 nm)	Interfere with virus attachment by binding with gp120 glycoprotein	[90]
Hepatitis B virus (HBV)	*Hepadnaviridae*	Silver nanoparticles (10 and 50 nm)	Reduce the formation of HBV DNA by binding with the HBV dsDNA and virions	[91]
Adenovirus type 3 (Ad3)	*Adenoviridae*	Silver nanoparticles (11.4 nm)	Damaging the viral particles and bind to the viral DNA	[92]
